# Hygiene of the First Dentition

**Published:** 1897-06

**Authors:** 


### Hygiene of the First Dentition.

Dr. Manuel Delfin, of Havana, presented a communication on the management of children during the first dentition. The question of main importance is that of the manner of feeding the infant, its health, especially at this trying period, depending in very great measure upon the character of the food upon which it is nourished. In decreasing order we find that the chief danger exists for those children whose alimentation consists entirely of artificial foods—the so-called infants' foods—which consist of elements unsuited to the digestive organs of young children. Secondly, we have children who are partly breast-fed and partly nourished artificially; these receive some food at least which they can digest, and receiving less artificial food, the stomach and intestines have less work to do in getting rid of this inert or often toxic material. Thirdly, those children who are wet-nursed, while best protected, are those who are fed entirely on their own mother's milk. In studying the hygiene of dentition we have to consider the infant at three periods of life: 1. From the date of its birth to that of the appearance of the first incisors. 2. During the actual period of the eruption of the teeth; and 3. During the period of weaning. The author then took up specially the subject of feeding, giving rules for the avoidance, as far as possible, of the evils attendant upon artificial feeding, and touching also upon hygiene of nursing, and the care of the child immediately after dentition and during the period of weaning.—*Pediatrics.*

